# Major Peptides from Amaranth (*Amaranthus cruentus*) Protein Inhibit HMG-CoA Reductase Activity

**DOI:** 10.3390/ijms16024150

**Published:** 2015-02-16

**Authors:** Rosana Aparecida Manólio Soares, Simone Mendonça, Luíla Ívini Andrade de Castro, Amanda Caroline Cardoso Corrêa Carlos Menezes, José Alfredo Gomes Arêas

**Affiliations:** 1 Faculty of Public Health, University of São Paulo, Av. Dr. Arnaldo, 715, São Paulo 01246-904, SP, Brazil; E-Mails: rosanaso@usp.br (R.A.M.S.); luila.andrade1@gmail.com (L.I.A.C.); amandacarlos@usp.br (A.C.C.C.C.M.); 2 EMBRAPA (Brazilian Corporation of Agricultural Research), PqEB, Brasília 70770-200, DF, Brazil; E-Mail: doutorasimone@gmail.com

**Keywords:** bioactive peptides, *de novo* sequencing, mass spectrometry, amaranth, HMG-CoA reductase activity

## Abstract

The objective of this study was to identify the major peptides generated by the *in vitro* hydrolysis of *Amaranthus cruentus* protein and to verify the effect of these peptides on the activity of 3-hydroxy-3-methyl-glutaryl-CoA reductase (HMG-CoA reductase), a key enzyme in cholesterol biosynthesis. A protein isolate was prepared, and an enzymatic hydrolysis that simulated the *in vivo* digestion of the protein was performed. After hydrolysis, the peptide mixture was filtered through a 3 kDa membrane. The peptide profile of this mixture was determined by reversed phase high performance chromatography (RP-HPLC), and the peptide identification was performed by LC-ESI MS/MS. Three major peptides under 3 kDa were detected, corresponding to more than 90% of the peptides of similar size produced by enzymatic hydrolysis. The sequences identified were GGV, IVG or LVG and VGVI or VGVL. These peptides had not yet been described for amaranth protein nor are they present in known sequences of amaranth grain protein, except LVG, which can be found in amaranth α‑amylase. Their ability to inhibit the activity of HMG-CoA reductase was determined, and we found that the sequences GGV, IVG, and VGVL, significantly inhibited this enzyme, suggesting a possible hypocholesterolemic effect.

## 1. Introduction

Amaranth seed is a barely known and underutilized grain, more commonly produced in Mexico, Peru and other Andean countries, and cultivated on a small scale in other countries from South America and Central America. Its production presents great potential and has been initiated around the world, mainly because the amaranth plant presents many interesting characteristics, such as fast growth (in about ninety days), the possibility of its use in grain crop rotation systems, and the high nutritional value of its leaves and seeds. The amaranth seed is high in protein (17%), and its amino acid composition is close to the optimum amino acid balance required in the human diet [1,2,3].

Besides these nutritional characteristics, studies have shown that introduction of amaranth seeds in the diet may prevent or diminish the risk of occurrence of diseases by presenting several biological activities [4,5,6,7].

Maier *et al.* [8] verified that fasting serum total cholesterol decreased 4.5% after a 28‑day dietary intervention in humans that consumed 50 g/day of amaranth. Mendonça *et al.* [4] tested the effect of the whole seeds or the protein isolated from amaranth in hamsters. The study indicated that amaranth protein reduced the cholesterol concentration in plasma, being the main component responsible for the hypocholesterolemic effect.

The unknown mechanisms by which amaranth proteins lower blood cholesterol levels have been investigated. Mendonça *et al.* [4] attributed this biological activity to peptides generated by an uncompleted digestion of the amaranth protein.

Small amounts of peptides and proteins, as well as some inert particles, can cross *in vivo* the normal small intestine in an intact form [9]. Research in humans has shown that peptides of different sizes, but with the *C*-terminus and *N*-terminus groups blocked, can pass through the intestinal epithelium [10,11]. After their absorption, these peptides may be transported through the cardiovascular system, interacting with tissues and organs where they exert their biological properties. Despite the intracellular hydrolysis, about 10% of the portal blood amino nitrogen is in the form of peptides [12].

Depending on the sequence of amino acids, the peptides produced in uncompleted digestion can exhibit diverse activities, including opiate-like, immunomodulatory, antimicrobial, antioxidant, antithrombotic, antihypertensive and hypocholesterolemic actions. Many of the known bioactive peptides are multifunctional and can exert more than one of the mentioned effects [13,14,15,16].

The studies about peptides encrypted in amaranth proteins, analyzed different species and used diverse enzymes to digest the proteins, reporting Angiotensin-Converting Enzyme (ACE) inhibitors and potential anticarcinogenic peptide actitivities [17,18,19,20]. Neither refers to the *Amaranth cruentus* species nor to the hypocholesterolemic effect. Thus, the objective of this study was to investigate the effect of the peptides generated by the *in vitro* hydrolysis of *Amaranthus cruentus* protein on the HMG-CoA reductase activity, and to identify these peptides.

## 2. Results and Discussion

### 2.1. Results

The preliminary tests with the two hydrolysis procedures and the two ways of interrupting the enzyme action showed that after ten minutes in all cases the hydrolysis degree does not change. The multi-enzyme method proposed by Hsu [21] was more effective in peptide recovery and the 10% trichloroacetic acid (TCA) addition for halting hydrolysis was the preferred method as it allowed a better separation of the remaining undigested protein (data not shown). All subsequent analyses were performed in triplicate in both digestion methods and the two interrupting processes with similar results, being shown only for the ones from the multi-enzyme method with TCA interruption.

Identification of the most abundant peptide components in protein hydrolysates involved the search for the masses and partial sequences (sequences tags) in the databases BIOPEP [22]; Pepbank [23] and several articles.

**Figure 1:**
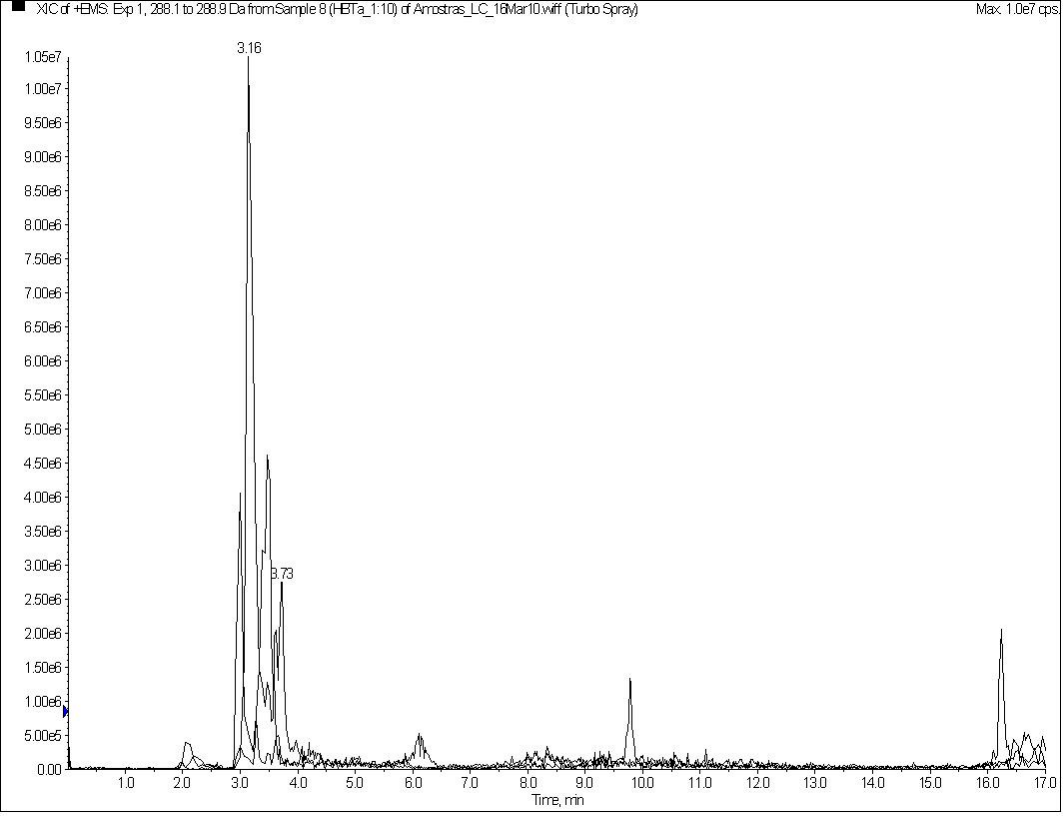
LC-ESI MS/MS chromatogram showing the major components in the *M*_r_ 3000 permeate after a multi-enzyme hydrolysis of amaranth protein isolate.

**Figure 2:**
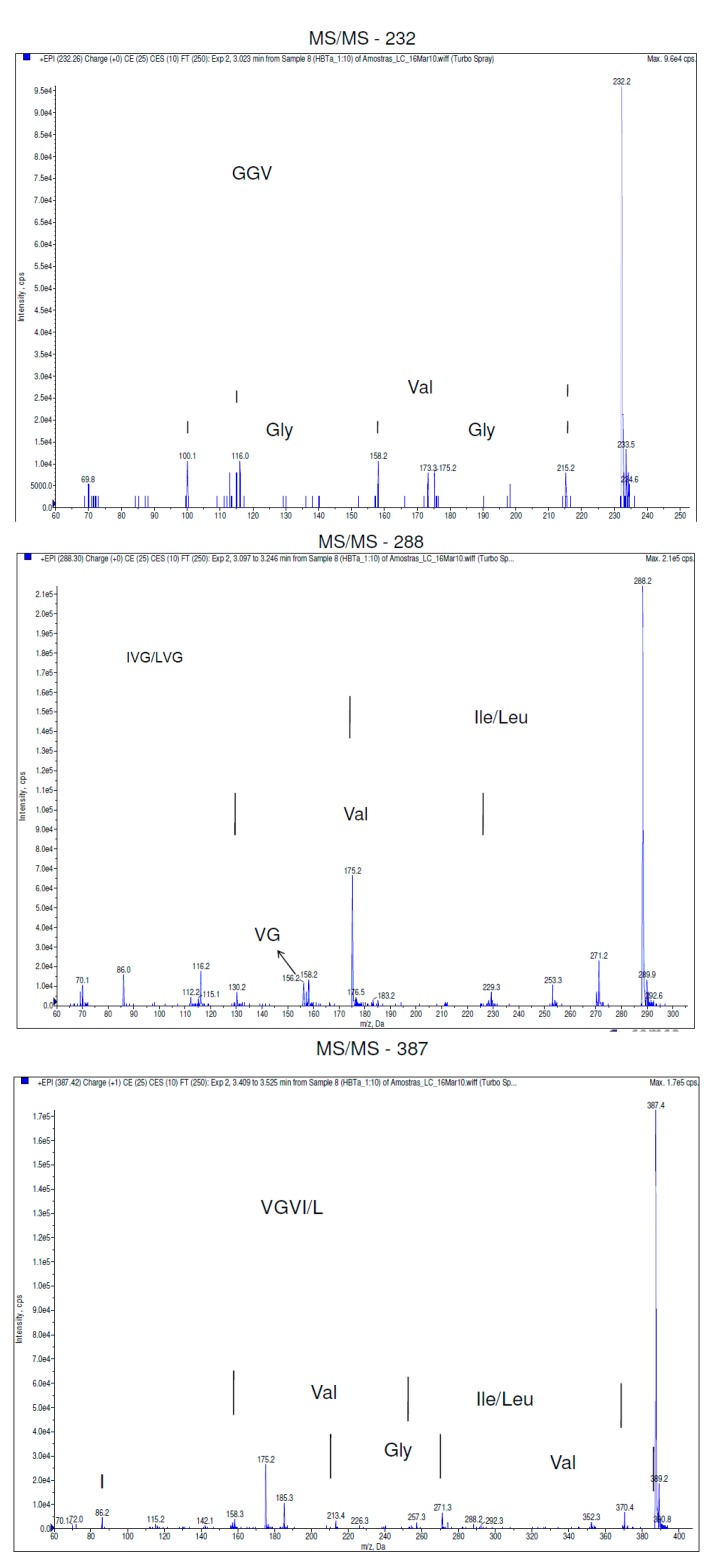
Mass spectrum of the selected chromatographic peaks (*m*/*z* 232, *m*/*z* 288, and *m*/*z* 387).

Figure 1 shows the peptide profile observed in the hydrolyzed samples. The three major peaks were chosen to be identified by the *de novo* technique. The difference between the fragments a (*m*/*z* 232), b (*m*/*z* 288) and c (*m*/*z* 387) and its subsequent ions resulted in Δ = 1. These indicate the presence of a unique charge on their masses, meaning that 232, 288 and 387 are the molecular ions of these peptides. MS/MS analysis provided the sequences *y* and *b*, from *C-*terminus (*y*) and *N-*terminus (*b*). The molecular ions (*m*/*z* 232, 288, and 387) were fragmented, resulting in the sequences shown in Figure 2.

The peptide (*m*/*z* 232) was identified as the sequence GGV. To the peptide (*m*/*z* 288) it was assigned the sequences IVG or LVG. The differentiation between L and I was not possible because of their identical masses. The tetrapeptide (*m*/*z* 387) was identified as VGVI or VGVL. Again, the differentiation between L and I was not possible because of their identical masses.

**Figure 3:**
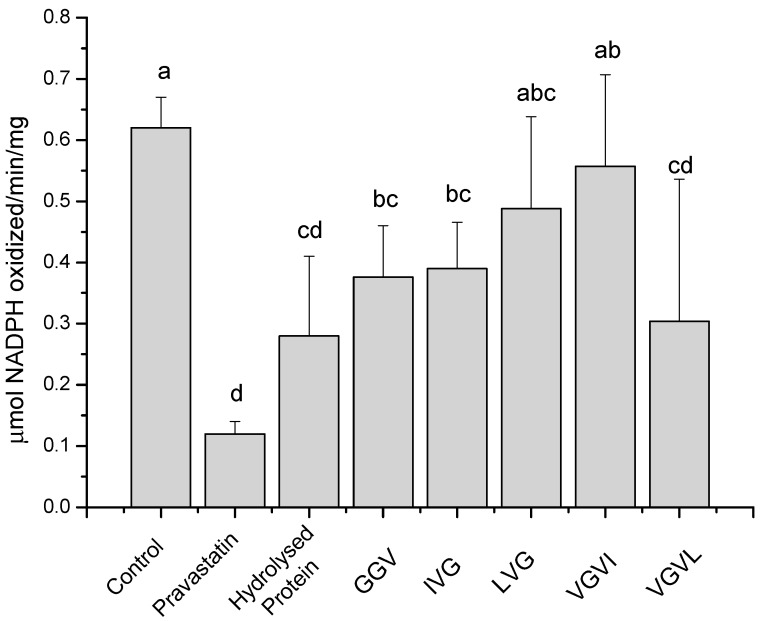
Evaluation of HMG-CoA reductase activity in control (absence of peptides or drugs), in positive control (in the presence of pravastatin), in hydrolyzed amaranth protein filtered through a 3 KDa *M*w cut off membrane, and in added of each peptide. Different letters mean significant differences among samples (*p* < 0.05).

The five peptides identified were synthesized by Aminotech Research and Development (Diadema, SP, Brazil), and their structure checked by (MS/MS spectrometry). They were then used in assays to verify their ability to inhibit HMG-CoA reductase. A control with the hydrolyzed protein isolated, filtered through the 3 KDa molecular weight cut off membrane was also performed, and the results are shown in Figure 3. This figure shows the significant inhibitory effect of the collection of peptides below 3 KDa. All tests were performed at 4 μg/mL concentration. The peptides VGVI and LVG have no significant effect in the HMG‑CoA reductase activity assay. In contrast, GGV, IVG, and VGVL compounds significantly inhibited the specific activity of HMG‑CoA reductase (Figure 3a).

### 2.2. Discussion

We followed the degree of hydrolysis and the peptides’ profile of both procedures tested and verified that the chosen multi-enzyme method [21], followed by TCA inhibition of digestion, resulted in higher quantity of peptides with a high or intermediate weight. A search in the protein sequences described for amaranth grain (Swiss Protein Data Bank, Protein Data Bank, GENBank) showed only ten proteins: agglutinin, alpha-amylase, alpha-amylase inhibitor, antimicrobial peptide precursor AMP2, globulin 11S, NAD-dependent malic enzyme precursor, non-specific lipid transfer protein, seed protein AmA1, trypsin inhibitor, and trypsin-subtilisin inhibitor. The detected sequences GGV, IVG, VGVL, and VGVI were not found when these ten protein sequences are submitted to *in silico* hydrolysis by trypsin, and the sequence LVG was found in alpha amylase (data not shown). As the sequences identified in this work were the major components under 3 KDa molecular size, other proteins in this grain should be sequenced to trace the origin of these peptides.

The search for biological action of the identified major peptides indicated that there is no specific function attributed to the peptide GGV. In the BIOPEP database [22], it was registered that this fragment is a sensorial peptide, classified as a bitter peptide. In fact, *Amaranthus cruentus* presents a bitter taste. As the concentrations of the four triterpene saponins present in *Amaranthus cruentus* seeds, which are the only other bitter compounds ever found in this species, are low (0.09%–0.1% of dry matter) [24], probably this peptide is one of the elements that confers this characteristic taste to the grain. The sequence GGV is also present in fragments containing higher molecular masses and that exhibit several biological activities, such as the red blood cells stimulating peptide (GGVYACRMGPITWVCSPLGG) [23].

The peptides LVG or IVG occur in other sequences with biological activity. According to BIOPEP Database [22], the sequences LLPIVGNLLKSLL~, LLPIVGNLLKSLLG, LLPIVGNLLNSLL~ and LLPIVGNLLNSLLG presented an antimicrobial activity. Peptides IVGRPRHQG and IVGRRRHQG are described as angiotensin‑converting enzyme inhibitors. All of the latter contain IVG in its sequence. There was no report in the literature about the biological activity of VGVL.

SILVA-SÁNCHEZ *et al.* [17] found in amaranth, diverse peptide residues in their work, such as IR, GKP, LF, FP, YL, RF, HY, VY and VW. There are several reasons for the differences observed in relation to our work. They worked with a different species, whose proteins may have distinct primary sequences, and they concentrated in the identification of peptides on the globulin fraction, whereas we worked with all proteins of the grain. Additionally there may be differences due to the use of the distinct *in vitro* hydrolysis method.

To date, there is no report of the peptides described in this paper for any of the amaranth species. Moreover, none of the peptides GGV, LVG, IVG, VGVL had already been described as hypocholesterolemic. The peptides LPYP and LPYPR were described as inhibitors of HMG-CoA reductase [25]. DVDPR and DPR were reported as inhibitors of lipid absorption. [26] IIAEK was described as reducing the micellar solution of cholesterol in the gut [27].

HMG-CoA reductase is a rate-limiting enzyme in cholesterol biosynthesis and is commonly targeted for drugs capable of a cholesterol-lowering effect [28]. Statins, such as pravastatin, are competitive inhibitors that interact at the HMG-CoA reductase binding site because they share a group that resembles the HMG portion of the enzyme, preventing the substrate from binding the enzyme by a steric mechanism. Since certain peptides are structurally unrelated to existing statins, the bioactive peptides found in this work might represent a novel class of HMG-CoA reductase inhibitors that can directly interact with this enzyme to block the mevalonate pathway and prevent hypercholesterolemia.

The *in vitro* inhibition of the HMG-CoA reductase by GGV, IVG, and VGVL differed from that of the control by distinct ranges. GGV and IVG present around 40% inhibition, but they were less effective as pravastatin (~90%). VGVL, on the other hand, was the strongest HMG-CoA reductase inhibitor (~45%), being statistically similar to pravastatin when these data were analyzed all together. Comparing by ANOVA individually the inhibition capacity of each peptide with pravastatin and with the control, all of them, except VGVI, present similar inhibition capacity.

The peptides GGV, IVG, and VGVL exhibit enough inhibition activity to be regarded as potential hypocholesterolemic bioactive peptides. The regular consumption of amaranth proteins results in effective cholesterol-lowering, which may be chiefly caused by the incomplete digestion, liberation and absorption of these peptides. More studies are necessary in order to verify the type of HMG-CoA reductase inhibition promoted by the peptides found in this study, if there is a synergic effect of their simultaneous consumption, and if they exert long-term hypocholesterolemic effects.

## 3. Experimental Section

### 3.1. Material and Reagents

Amaranth (*Amaranthus cruentus*) grain was obtained from Embrapa Cerrados (Planaltina, DF, Brazil). Hexane used for sample preparation was purchased from Dinamica (Diadema, SP, Brazil). Hydrogen chloride, potassium hydroxide, potassium sulfate, copper(II) sulfate, sulfuric acid, boric acid, citrate and phosphate buffer, trichloroacetic acid were of analytical grade and were obtained from Casa Americana (São Paulo, SP, Brazil). Acetonitrile (HPLC grade) were purchased from Carlo Erba (Rodano, Italy). Pepsin (P6887, 3200–4500 units/mg protein), tripsin (T-0303, 17,600 U/mg of protein), α-quimotripsin (C-4129, 54 U/mg of protein) and peptidase (Sigma P7500, 102 U/g of solids) were purchase from Sigma (St. Louis, MO, USA). Synthetic peptides were obtained from Aminotech Research and Development (Diadema, SP, Brazil) and presented a purity degree above 99%. HMG-CoA Reductase Assay Kit was purchased from Sigma (CS1090).

### 3.2. Sample Preparation

#### 3.2.1. Protein Isolate Preparation

The amaranth protein isolate (API) was prepared from hexane defatted amaranth (*Amaranthus cruentus*) flour as described by Arêas [29], with protein extraction at pH 11 and subsequent isoelectric precipitation at pH 5.7, according to Gueguen *et al.* [30] and Mendonça *et al.* [4]. An aqueous dispersion of this flour (1:5, *w*/*v*) was brought to pH 11.0 with 1 mol·L^−1^ NaOH, and after mixing for 4 h at 25 °C, was then centrifuged at 9000× *g* for 20 min, at 4 °C. The supernatant fraction was adjusted to pH 5.7 with 1 mol·L^−1^ HCl and centrifuged again. Finally, the protein pellet was resuspended in water (1:5, *w*/*v*), the pH was adjusted to 7.0 with 1 mol·L^−1^ NaOH, and then lyophilized.

The resulting powder was submitted to further defatting with ethanol to increase the purity of the isolate. Protein was analyzed by the micro-Kjeldahl method and moisture was determined by a gravimetric method [31]. The analyses were performed in triplicate.

#### 3.2.2. Protein *in vitro* Digestion

We tested the *in vitro* digestion of the amaranth protein isolate (API) by two different methods.

The first was a two-stage method developed by Qiao *et al.* [32]. In brief, in Stage 1, pepsin (120 U/mL, or 0.25% enzyme protein relative to substrate protein) was used to digest the substrate proteins (12.5 mg/mL) in citrate buffer solution (pH 2) for 24 h. For Stage 2, phosphate buffer solution for a final pH 8 and trypsin-enriched pancreatin (activity equivalent to at least three U.S. Pharmacopeia/mL, or 7.5% enzyme protein relative to substrate protein, final substrate concentration 5 mg/mL) were added, and the digestion continued for additional 96 h. Incubations were carried out at 38 °C under continuous agitation.

The second *in vitro* assay was performed by an enzymatic method according to Hsu [21], where 250 mg of the amaranth protein isolate was added to a multi-enzyme system consisting of trypsin (5.16 mg), α-chymotrypsin (13.76 mg) and peptidase (1.28 mg). The enzymes and substrate were dissolved in 40 mL of phosphate buffer (pH 8.0) and kept at 37 °C under continuous agitation for ten minutes.

For both methods, the digestion was interrupted by adding 10% *w*/*v* trichloroacetic acid or by cooling the tubes, resulting in four different samples, which were independently carried out in triplicate. Samples were centrifuged at 1150× *g* for fifteen minutes, and the supernatant was used for the following analyzes, which are described next. After comparing the yield and the peptide profiles of all samples, we chose the multi-enzyme method [21] with hydrolysis interruption by 10% TCA, which gave more reproducible results.

### 3.3. Analyses

#### 3.3.1. Estimation of Soluble Nitrogen

Ten milliliters of each digested supernatant was analyzed for nitrogen by the micro-Kjeldahl method [31]. The net increase in soluble nitrogen was calculated after taking into account the corresponding blank values.

#### 3.3.2. Peptides Profile

Fifty microliters of the digested supernatant were ultrafiltered on a hydrophilic *M*_r_ 3000 cut-off membrane (Centripep, Amicon, Beverly, MA, USA). The permeates were freeze-dried and kept at −20 °C until use. The peptides were extracted with acetonitrile, dried, and redissolved in 100% mobile phase A for HPLC analysis.

RP-HPLC separation of the *M*_r_ 3000 permeates was performed on a Shimadzu HPLC system (LC10AT, Nakagyo-ku, Kyoto, Japan). The HPLC system was equipped with a quaternary gradient pumping system, an in-line degasser, a variable-wavelength absorbance detector set at 220 nm, and a Rheodyne injector. The column used in these experiments was a 250 mm × 4.6 mm Atlantis C18 column (Waters, Milford, MA, USA). The injection volume was 20 µL.

Solvent A was a mixture of water, trifluoroacetic acid and acetonitrile (98.925:0.075:1.000, *v*/*v*/*v*) and solvent B contained acetonitrile, water and trifluoroacetic acid (69.935:30.000:0.065, *v*/*v*/*v*). Peptides were eluted with a linear gradient of 0%–100% solvent B over 60 min at a flow rate of 0.5 mL/min. The software used to process the chromatograms was the Class VP.

#### 3.3.3. LC-ESI MS/MS Analyses

Fifty microliters of the digested supernatant were ultrafiltered on a hydrophilic *M*_r_ 3000 cut-off membrane (Centripep, Amicon, Beverly, MA, USA). The permeates were freeze-dried and kept at −20 °C until use. The peptides were extracted with acetonitrile, dried, and redissolved in 5% mobile phase B (in mobile phase A) for LC/MS/MS analysis.

LC-ESI MS/MS analyses of the permeates containing peptides with molecular masses below 3000 Da were performed on an LC-MS/MS system consisting of an Agilent 1200 series liquid chromatograph (Palo Alto, CA, USA) coupled with an Applied Biosystems 3200 QTRAP tandem mass spectrometer (Foster City, CA, USA).

The column used in these experiments was a Vydac Protein and Peptide C18, 150 mm × 2.1 mm (Vydac, Hesperia, CA, USA). The injection volume was 10 µL. Solvent A was a mixture of water and formic acid (100:0.1, *v*/*v*) and solvent B contained acetonitrile and formic acid (100:0.1, *v*/*v*). Peptides were eluted initially with 95% A and 5% B mobile phase, this proportion was maintained for 3.5 min. After that, a gradient of solvent B in A went from 5%–40% in 8.5 min and solvent B in A went from 40%–90% in 3.0 min. This final proportion was kept until the end of the analysis for two more minutes. The flow rate was 400 nL/min.

Synthetic air was used as nebulizer gas for the electro-spray ionization. The capillary was held at 5.5 kV. Spectra were recorded over the mass/charge (*m*/*z*) range 150–700. After Full Scan, the Enhanced Product Ion (EPI) of the most abundant peaks was analyzed, generating its fragmentation. Spectra of each one of these ions were submitted to *de novo* sequencing.

#### 3.3.4. HMG-CoA Reductase *in vitro* Assay

The HMG-CoA reductase assay kit from Sigma-Aldrich (St. Louis, MO, USA) was used under conditions recommended by the manufacturer. Reference statin drug—pravastatin—was used as positive control. In summary, aliquots containing 4 μL of NADPH (to obtain a final concentration of 400 μM) and 12 μL of HMG-CoA substrate (to obtain a final concentration of 0.3 mg·mL^−1^) were placed into an UV compatible 96 well plate. Phosphate buffer pH 7.4 was also added in order to achieve the final volume of 0.2 mL per well. The analyses were initiated (time 0) by the addition of 2 μL of the HMG-CoA reductase (concentration of the enzyme stock solution was 0.50–0.70 mg protein/mL) and incubated in at 37 °C in the presence or absence (control) of 1 μL aliquot of pravastatin or 4 μg of each peptide. The rates of NADPH consumed were monitored every 20 s for up to 600 s by reading the decrease in absorbance at 340 nm, using a SpectraMax M5 equipment (Molecular Devices, Sunnyvale, CA, USA). Results were expressed as μmol of NADPH oxidized/min/mg protein in the presence or the absence of drug or peptides.

## 4. Conclusions

In summary, our data have demonstrated that the *Amaranthus cruentus* protein encrypts the amino acid sequences GGV, IVG/LVG, VGVI/VGVL, and that these peptides represented the most abundant ones found. The peptides GGV, IVG, and VGVL exhibited an *in vitro* inhibition of the HMG-CoA reductase, suggesting its hypocholesterolemic effect. This is the first report of the occurrence of these peptides in amaranth grain protein and the first report of HMG-CoA reductase effect by the peptides GGV, IVG, and VGVL.
